# Editorial: The Role of the Microbiome in Regulating T-Cell Response in Asthma and Food Allergy

**DOI:** 10.3389/fimmu.2021.782720

**Published:** 2021-10-14

**Authors:** Ayşe Kiliç, Hani Harb

**Affiliations:** ^1^ Channing Division of Network Medicine, Department of Medicine, Brigham and Women’s Hospital and Harvard Medical School, Boston, MA, United States; ^2^ Laboratory of Psychoneuroimmunology, Institute for Psychosomatic and Psychotherapy, Justus-Liebig-University Giessen, Giessen, Germany; ^3^ Division of Immunology, Boston Children’s Hospital, Harvard Medical School, Boston, United States

**Keywords:** asthma, food allergy, microbiota, Treg - regulatory T cell, T-cell, microbial metabolites

The role and importance of the microbiome for human health has been investigated in recent years (1, 2). A dysbiosis of the gut microbiome has been shown to cause drastic changes in the immune system (3, 4) Leading to disbalance of immune homeostasis and consequently to the emergence of different diseases (5, 6). In this editorial, we investigate the role of microbiome in regulating immune response in Asthma and Food Allergy.

The interplay between gut and lung as two separate organs has been introduced previously (7–10). In their review, Di Gangi et al. have explored this concept and investigated the importance of gut microbiota in protection or augmentation of allergic asthma. Click or tap here to enter text.The authors shed light on the importance of *Lactobacillus spp* as an important part of the human gut microflora (11). Data from clinical cohorts hint towards a link between microbial dysbiosis and asthma risk in children. They review the findings from e.g. the PASTURE, EFRAIM and WHEALS cohorts. These studies show a protective effect of farm-exposure on asthma risk in children early in life with higher Treg numbers in peripheral blood of children consuming farm-milk (12, 13). Key findings of the WHEALS study include a delayed diversification of the gut microbiota and a relative difference in the composition with fewer *Lactobacillus, Bifidobacterium, Akkermisia* and *Faecalibacterium* and more *Candida spp* (14). The protective effect, however, was not lasting. The reasons for this phenomenon are not understood.

Collectively, there has been much research focusing on the role of microbiota in the development of asthma. The “Hygiene hypothesis” has put a lot of value on how bacterial species and bacterial metabolites are protective against asthma and allergy development. In that sense, Hagner and Harb et al. showed a protective effect of different microbial species, either isolated from cow shed or used as prebiotics, against the development of different hallmarks of allergic airway inflammation in mice (11, 15). These protective effects were transferred from mothers to offspring *via* TLR activation and signaling in the mothers exposed to these bacterial species (16). Mechanistically, these protective effects were related to epigenetic modification of the IFNG promoter in T-cells (17).

Bacterial fermentation in the lower gastrointestinal tract degrades indigestible complex carbohydrates from fiber, amino acids or mucus and gives rise to a variety of microbial metabolites. These metabolites include hydrogen; organic acids, such as lactate and succinate; alcohols, such as 1,2 propanediol; and short-chain fatty acids (SCFAs), such as acetate, butyrate, formate, propionate, and pentanoate [reviewed in (18)]. With highest concentrations in the gut (within millimolar range), SCFAs are transported *via* proton-coupled monocarboxylate transporter isoform 1 (MCT1, gene name SLC16A1) or the Na^+^-coupled monocarboxylate transporter 1 (SMCT1, gene name SLC5A8) into colonocytes where a large part is metabolized locally for energy production (19, 20). Only ow levels reaching the blood circulation and therefore peripheral organs, it is assumed that SCFAs interact with gut resident immune cells, which then affect immune processes in peripheral tissues. In that regard, Yip et. al. review the expression of butyrate sensing cell-surface receptors of the G protein-coupled receptor (GPCR), namely GPR41, GPR43, and GPR109A, on leukocyte subsets and downstream regulated cellular mechanisms. The broad anti-inflammatory activity of SCFAs is achieved by either activating the Peroxisome proliferator-activated receptors, like PPARγ1, or inhibiting histone deacetylases (HDACs) activity and therefore promoting gene transcription from targeted chromatin.

While the beneficial effect of SFCAs in human disease is currently unclear, mouse models of ovalbumin- and house dust mite-induced allergic airway inflammation provide positive results. Oral administration of high fiber diet or SFCAs in mice, either nursing dams or uptake *ad libitum*, alleviated the symptoms in allergic airway inflammation, including airway reactivity, systemic immunization, and leukocyte infiltration into the lung. *Ex vivo* experiments, reviewed by Luu et al. and Yip et. al., in this Research Topic, highlight the diverse levels of regulation by SCFAs in general and only butyrate. Butyrate exposure reduces dendritic cell activation and migration to local lymph nodes, thereby limiting Th2 polarization of naive CD4+ T cells post allergen exposure. Under Th9 polarizing conditions, butyrate induces Foxp3^+^ expression and therefore enforces a regulatory phenotype (21, 22). In ILC2 the secretion of IL-5 and IL-13, with pronounced downstream effects on eosinophils, was suppressed. Butyrate further reduces isotype/class switching in B cells reducing IgE production (23).

On the other hand, Zakzuk et al. explored how helminth infection shapes the landscape of the T-cell immunity in patients with asthma. In their original article, the authors collected peripheral blood mononuclear cells from helminth infected and healthy control subjects in the rural areas of Colombia. They report an inverse correlation between egg worm burden and histone 4 (H4) acetylation at the IL-13 gene. Additional results comprise a significant correlation between the same histone acetylation mark at the IL-4, CHI3L1 genes and IgE levels to *Ascaris lumbricoides*. In relation to asthma, there were significant associations between HDM specific IgE antibodies and H4-acetylation levels in the gene *TNFSF13B* encoding the B cell activating factor (BAFF).

Finally, in our proposal for this Research Topic, it was our intention to gather ideas, research, thoughts, and insights into the role of microbiota in regulating T-cell responses in different allergic diseases. Unfortunately, our topic stumbled by the beginning of the pandemic, closure of laboratories and stay at home orders in different countries. We believe that this topic carries an extensive potential between its pages and it would be great, if the future focus of allergy/microbiota research would still be on regulating different immune processes in the hosts.

## Author Contributions

All authors listed have made a substantial, direct, and intellectual contribution to the work and approved it for publication.

## Funding

For HH: German Research Foundation (DFG) grant Nr. HA 8465/1-1 and For AK: Research Foundation (DFG) grant Nr. KI 1868/3-1.

## Conflict of Interest

The authors declare that the research was conducted in the absence of any commercial or financial relationships that could be construed as a potential conflict of interest.

## Publisher’s Note

All claims expressed in this article are solely those of the authors and do not necessarily represent those of their affiliated organizations, or those of the publisher, the editors and the reviewers. Any product that may be evaluated in this article, or claim that may be made by its manufacturer, is not guaranteed or endorsed by the publisher.
